# The effect of phage genetic diversity on bacterial resistance evolution

**DOI:** 10.1038/s41396-019-0577-7

**Published:** 2020-01-02

**Authors:** Jenny M. Broniewski, Sean Meaden, Steve Paterson, Angus Buckling, Edze R. Westra

**Affiliations:** 1 Biosciences, Environment and Sustainability Institute, University of Exeter, Penryn, TR10 9FE UK; 2 Institute of Integrative Biology, University of Liverpool, Liverpool, L69 7ZB UK

**Keywords:** Molecular evolution, Bacterial evolution, Bacteriophages

## Abstract

CRISPR-Cas adaptive immune systems are found in bacteria and archaea and provide defence against phage by inserting phage-derived sequences into CRISPR loci on the host genome to provide sequence specific immunological memory against re-infection. Under laboratory conditions the bacterium *Pseudomonas aeruginosa* readily evolves the high levels of CRISPR-based immunity against clonal populations of its phage DMS3vir, which in turn causes rapid extinction of the phage. However, in nature phage populations are likely to be more genetically diverse, which could theoretically impact the frequency at which CRISPR-based immunity evolves which in turn can alter phage persistence over time. Here we experimentally test these ideas and found that a smaller proportion of infected bacterial populations evolved CRISPR-based immunity against more genetically diverse phage populations, with the majority of the population evolving a sm preventing phage adsorption and providing generalised defence against a broader range of phage genotypes. However, those cells that do evolve CRISPR-based immunity in response to infection with more genetically diverse phage acquire greater numbers of CRISPR memory sequences in order to resist a wider range of phage genotypes. Despite differences in bacterial resistance evolution, the rates of phage extinction were similar in the context of clonal and diverse phage infections suggesting selection for CRISPR-based immunity or sm-based resistance plays a relatively minor role in the ecological dynamics in this study. Collectively, these data help to understand the drivers of CRISPR-based immunity and their consequences for bacteria-phage coexistence, and, more broadly, when generalised defences will be favoured over more specific defences.

## Introduction

Bacterial and archaeal viruses can influence microbial communities both through direct interactions with their hosts as well as indirect effects on non-host populations [1–4]. The selective pressure of viral predation has resulted in the evolution of a range of archaeal and bacterial defence mechanisms, which includes CRISPR-Cas (clustered regularly interspaced short palindromic repeats; CRISPR-associated) systems, prokaryotic argonautes, restriction–modification (R–M) systems, surface modification (sm), toxin–antitoxin and abortive infection systems [5]. These defences differ in multiple ways including the range of phage to which they provide resistance (generalist versus specialist) and in their fitness costs [6]. In bacteria two of these mechanisms, sm and CRISPR-Cas systems, are often found to be important for rapid evolution of phage resistance, with sm perhaps representing the simplest, and the most common, line of defence by mutation of the bacterial cell surface, obscuring or altering the receptor that is used by the phage to attach to the cell [7]. Alternatively, bacteria may employ their CRISPR-Cas adaptive immune systems, which occur in ~30% of the bacterial genomes sequenced to date [8]. Initially naive bacteria acquire CRISPR-based immunity by incorporating nucleic acids from phage genomes into CRISPR loci on their own genome [9, 10], where they are known as “spacers” [11]. Processed CRISPR transcripts guide Cas proteins to detect and destroy re-infecting phage that carry the cognate target sequence [12, 13]. Phage can in turn overcome CRISPR-based immunity by acquiring a point mutation in the target sequence on their genome [14], although the emergence and spread of such phage “escape” mutants is constrained by both the diversity of CRISPR immunity alleles in the bacterial population and the fitness costs associated with the phage mutations [15–18].

While our mechanistic understanding of the different defence strategies of bacteria has raced ahead, the ecological conditions that favour one type of defence over another, and their consequences for the ensuing bacteria-phage population dynamics remain unclear [6, 19]. Previous work has shown that the inducible cost of CRISPR-Cas means CRISPR-based immunity will typically be favoured over more costly constitutive defences, such as sm, under relatively low forces of infection [20]. However, because the cost of sm-based resistance is fixed, it may provide a selective advantage when the force of infection is high. Given that the cost of sm-based resistance can increase in the presence of bacterial competitors, CRISPR-based immunity evolution may generally be more important in a microbial community context [21].

Here, we focus on another key distinction between the different types of defence: their different levels of specificity. CRISPR-Cas mediated defence is necessarily fairly specific, with a single spacer unlikely to be able to target all phage genotypes within a population. While spacer diversity within bacterial populations provides an additional population level resistance by increasing the chance of phage extinction, this benefit may be reduced if phage populations are sufficiently diverse and hence there are variants that can escape targeting by many spacers. By contrast, sm-based resistance provides resistance against a broad range of phage genotypes, and typically requires multiple mutations for phage to be overcome [22–24]. We therefore hypothesised that increasing the diversity of infecting phage populations would increase selection for sm-based resistance.

Here we test the effect of phage genetic diversity using *P. aeruginosa* PA14 and its phage DMS3vir. *P. aeruginosa* is an opportunistic human pathogen and a model organism for studying the mechanism and evolutionary ecology of CRISPR-Cas systems. *P. aeruginosa* PA14 can evolve resistance to the phage DMS3vir through either sm or CRISPR-Cas [20, 25]. In order to directly test the impact of phage genetic diversity on the frequency at which CRISPR-based immunity evolves, we generated phage DMS3vir populations with increased genetic diversity by serial passage of the phage on a phage-sensitive *P. aeruginosa* PA14 derived strain. We then exposed the bacterium *P. aeruginosa* strain PA14 to the genetically more diverse populations of the phage DMS3vir, or to a clonal phage DMS3vir population. Following infection with genetically diverse phage populations, a smaller proportion of the initially phage-sensitive bacterial populations relied on CRISPR-based immunity. Moreover, those bacteria that relied on CRISPR-based immunity in the face of diverse phage populations acquired more spacers per individual compared with those that were exposed to clonal phage populations, presumably because this allows them to resist a wider range of phage genotypes [16, 26]. The ultimate effect of the frequency of CRISPR-based immunity on the rate of phage extinction was however independent of the levels of standing genetic variation in the phage populations.

## Methods

### Bacterial strains and phages

*P. aeruginosa* UCBPP-PA14 (referred to as WT, carrying no spacers with a perfect match to the DMS3vir genome), *P. aeruginosa* UCBPP-PA14 *csy3::LacZ* [25] (referred to as CRISPR-KO, since it carries a disruption of an essential *cas* gene that causes the CRISPR-Cas system to be non-functional), and *P. aeruginosa* UCBPP-PA14 *mutS*::MAR2xT7 [27], which was kindly provided by Alexandro Rodriguez Rojas (below this strain is also referred to as a mutator strain of PA14), and the CRISPR-KO-derived sm (described previously in ref. [20]), were used in all experiments. The obligately lytic phage DMS3*vir* was used in all experiments, and has previously been described in Cady et al. [25]. All statistical analyses were carried out in R version 3.4.4.

### Generating diversity within phage populations

To generate genetically diverse populations of phage DMS3vir, the phage was amplified on *P. aeruginosa* UCBPP-PA14 *mutS*::MAR2xT7, a transposon mutant of *P. aeruginosa* that lacks the *mutS* gene. No DNA-polymerase has been characterised on the DMS3vir genome so we hypothesised that replication on a mutator strain of its host would result in increased levels of genetic variation within the phage population. Bacteria and phage were grown in 10 ml LB media, by inoculating 1:100 from an overnight culture of *P. aeruginosa* UCBPP-PA14 *mutS*::MAR2xT7 and subsequent infection with 10^6^ pfu (plaque forming units) of DMS3vir. Twelve independent cultures were inoculated, followed by incubation at 37 °C while shaking at 180 rpm. After 24 h phages were sampled via chloroform extraction by mixing the overnight cultures 2:1 with chloroform followed by vortexing and centrifugation at 3500 rpm to pellet bacterial debris. Supernatant containing phage was stored at 4 °C and used to infect naive *P. aeruginosa* UCBPP-PA14 *mutS*::MAR2xT7 cells in fresh media, as described above. This procedure was repeated daily for 17 days, resulting in phage populations with increased levels of genetic diversity. We then generated derived clonal populations by isolating a single phage clone from each of the twelve diverse populations, using double plaque purification, followed by a single round of amplification on *P. aeruginosa* UCBPP-PA14 *csy3::LacZ* bacteria.

### Deep sequencing of phage populations

To measure the levels of standing genetic variation in the diverse and derived clonal phage populations that were generated as described above, we extracted phage DNA from ~10^8^–10^9^ pfu using the Norgen phage DNA isolation kit, following the manufacturer’s instructions. After QC with Nanodrop, Qubit and electrophoresis to quantify the amount and quality of extracted material, the extracted DNA was sequenced using MiSeq by the Liverpool Center for Genomic Research, using previously described protocols [16]. Ancestral virus was processed in parallel as a control for SNP calling. Barcoded Illumina Truseq Nano libraries were constructed from each DNA sample with an ~350 base insert size and 2 × 250 base reads generated on an Illumina MiSeq platform. Reads were trimmed for the presence of Illumina adaptor sequences using Cutadapt version 1.2.1 and Sickle version 1.200 with a minimum window quality score of 20. After trimming most reads were around 250 bases and reads shorter than 10 bases were removed. Overlapping reads were joined using Flash version 1.2.8 to create high quality sequence at ~8000× coverage of DMS3vir per sample. Reads from evolved phage populations were mapped to the ancestral DMS3vir genomes using bwa mem version 0.7.12. Sites which had coverage lower than 100 and an alternate allele frequency of >0.005 in the ancestor were filtered out to minimise sequencing error and noise. Data for SNPs which were fixed (>0.95 frequency) in any of the clonal population samples was extracted. No fixed mutations were found in the diverse populations that were not present in one of the clonal populations. Sequence data have been deposited in the European Nucleotide Archive under accession number ENA: PRJEB31472

### Infectivity of genetically diverse phage on CRISPR-resistant bacteria

We investigated the effect of phage genetic diversity on the ability of the phage population to overcome CRISPR targeting by testing the 12 experimentally evolved phage populations for infectivity against 12 CRISPR-immune bacteria. The bacteria used for infectivity tests were obtained from previous co-evolution experiments with DMS3vir. The selected isolates have been sequenced to ensure each was carrying a unique single spacer sequence [16]. The infectivity of phage was determined by spot assay on a lawn of each of the CRISPR-immune bacteria and the CRISPR-KO strain (which carries an inactive CRISPR-Cas system and is therefore sensitive to phage infection). Infectivity is expressed as the efficiency of plaquing (EOP) on the CRISPR-immune host, which was calculated by dividing the pfu/ml formed by the phage population on the relevant CRISPR-immune bacteria by the pfu/ml formed on the CRISPR-KO strain. In addition, we tested all phage populations’ infectivity against 6 CRISPR-immune bacterial strains each carrying two spacers, and against one strain with sm-based resistance.

### Measuring the effect of diversity on phage persistence

To investigate how genetic diversity within a phage population affects the phage population dynamics we inoculated 6 ml M9 media supplemented with 0.2% glucose 1:100 with WT cells and added 10^4^ pfu of either clonal or diverse phage. Phage titres were measured after 1, 3 and 5 days: Phages were extracted using chloroform as described above and a dilution series was spotted on a lawn of soft LB (0.5% agar) inoculated with *P. aeruginosa* PA14 *csy3::LacZ* to quantify phage titres.

### Effect of phage diversity on CRISPR-based immunity evolution

To investigate how the diversity of an infecting phage population affects the evolution of resistance mechanisms in the host we co-cultured WT *P. aeruginosa* UCBPP-PA14 with either clonal or diverse phage for 3 days. Glass vials were filled with 6 ml of M9 media supplemented with 0.2% glucose and inoculated with 10^6^ cfu/ml of *P. aeruginosa* and 10^4^ pfu of phage from one of either the diverse or clonal populations (*n* = 8) then incubated for 24 h (±3 h) at 37 °C while shaking at 180 rpm. Transfers to fresh media were performed daily at a concentration of 1:100. The bacterial populations were sampled at 3 dpi (days post infection) and resistance profiles of the bacterial isolates from the experimental populations were determined via streak assays against either ancestral phage (DMS3*vir*) or the isogenic phage DMS3*vir*-AcrIF1, which carries an antiCRISPR (Acr) gene that blocks the CRISPR-Cas system of *P. aeruginosa* UCBPP-PA14 [28]. Lines of phage were applied to agar plates and allowed to dry for 20 min. Bacterial colonies were streaked across the lines of phage and plates were incubated overnight at 37 °C. Bacteria were scored to be CRISPR immune when they were resistant to DMS3*vir*, but not to DMS3*vir*-AcrIF1 (also associated with a motile, swarming phenotype) and surface-based resistance was scored when they were resistant to both phages (also associated with a smooth, non-motile phenotype). Bacteria were scored to be sensitive when they displayed resistance to neither phage. In addition, evolution of CRISPR-based immunity was further confirmed by PCR of both CRISPR loci, using primer pairs 5′-CTAAGCCTTGTACGAAGTCTC-3′ and 5′-CGCCGAAGGCCAGCGCGCCGGTG-3′ to determine spacer acquisition at the CRISPR 1 locus, and primer pairs 5′-GCCGTCCAGAAGTCACCACCCG-3′ and 5′-TCAGCAAGTTACGAGACCTCG-3′ to determine spacer acquisition at the CRISPR 2 locus.

## Results

### Diverse phage populations harbour more SNPs

Given the sequence specificity of CRISPR-based immunity against phage, we hypothesised that the phage genetic diversity will constrain the evolution of CRISPR-based immunity relative to sm-based resistance, with potential knock-on effects for phage persistence. To test this, we amplified phage DMS3vir on a mutator strain of *P. aeruginosa* PA14 with the aim of increasing phage genetic diversity. After 17 passages of DMS3vir on the mutator strain, we performed deep sequencing analysis on the phage populations to examine if the standing levels of genetic diversity (i.e. SNPs frequency) were increased relative to paired clonal control populations that were derived from each of the diverse populations.

**Fig. 1:**
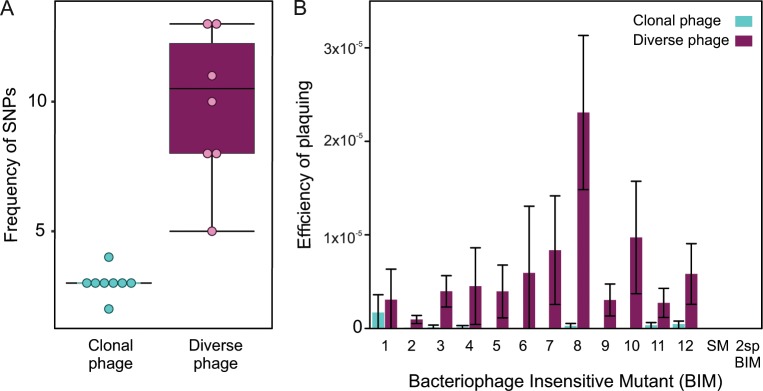
The 12 evolved phage populations harbour greater genetic variation than the derived clonal populations. **a** The frequency of SNPs in diverse (purple) phage populations (*N* = 12) and clonal (blue) (*N* = 12) populations. **b** The average efficiency of plaquing of 12 experimentally evolved diverse phage populations, or the 12 derived clonal phage populations on each of 12 CRISPR-resistant bacteriophage insensitive mutants (BIMs). Each BIM carries a single unique spacer targeting the ancestral phage. Infectivity of phage populations was also tested against six BIMs carrying two spacers targeting the ancestral phage (these data are shown as one bar as no escape mutants were identified against any of the six clones possessing two spacers each) and a surface mutant (sm). All graphs show averages and error bars represent 95% confidence intervals.

Although the sensitivity of deep sequencing is insufficient to identify rare genotypes, it revealed 40 SNPs across the 16 phage populations (eight “clonal” and eight “diverse”) which were present at 0.01% frequency or higher after processing to remove sequencing noise and error. All of the SNPs found in clonal populations were also present in the paired diverse population. Based on this analysis, diverse populations were found to contain an average of 9.2 SNPs (ranging from 5–13 in each population), while clonal populations were found to only have an average of three SNPs (ranging from 1–4). However, SNP frequencies based on deep-sequencing analysis inevitably underestimate the true level of genetic variation in the populations as rare alleles are filtered out to limit the noise from sequencing errors. Nonetheless, a Kruskal–Wallis test confirmed that the diversified phage populations had a higher average SNP frequency than the paired clonal strains (Fig. 1a) (*χ*^2^(2) = 11.94, *p* < 0.001), and most SNPs were located close to gene 42, which contains a CRISPR priming site [20], which suggests that the observed SNP clustering may result from acquisition of spacers from this area of the phage genome and subsequent evolution of escape by the phage during the passaging on the mutator strain.

### Genetically diverse phage are more infective on hosts with CRISPR-based immunity

Previous work has shown that point mutations in the phage genome sequence that is targeted by the CRISPR-Cas immune system often allows the phage to by-pass this defence [14, 16, 29, 30]. We therefore predicted that the genetically more diverse phage populations would have greater infectivity on bacteria with CRISPR-based immunity compared with their paired clonal phage populations or the ancestral population. Given that it is much harder for phage to evolve to recognise a novel receptor [24], we expected that the levels of infectivity of the diversified and clonal phage populations would be the same on a bacterium with sm-based resistance. To test these ideas, we examined the levels of infectivity for each of our diversified and clonal populations against 12 clones with CRISPR-based immunity of *P. aeruginosa* PA14, each possessing a single unique spacer, and against six clones with CRISPR-based immunity each carrying two spacers targeting DMS3vir, which is harder to overcome by point mutation [16, 26], as well as a PA14 strain with sm-based resistance. As expected, we found that infectivity of genetically more diverse phage populations was higher on bacteria with CRISPR-based immunity than that of clonal phage populations (Wilcoxon signed rank test, *W* = 1359, *Z* = −9.5082, *p* < 0.0001), but neither of the phage populations were infective against hosts with two spacers or sm-based resistance (Fig. 1b). Consistent with the idea that SNPs are present at a low frequency in the phage population, the EOP of more genetically diverse phage was typically around 10^−5^ for each spacer tested, an increase of around 10–200 fold compared with the clonal phage populations. Collectively, these data support the idea that phage diversified during the repeated amplification on the mutator strain, resulting in greater infectivity on CRISPR-resistant bacteria.

### Type of evolved host resistance depends on the levels of phage genetic diversity

**Fig. 2:**
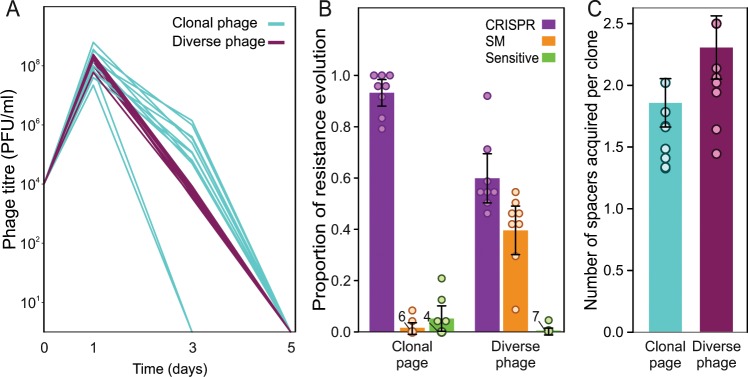
No effect of phage genetic diversity on phage persistence. **a** Phage titres (pfu/ml) over time following infection of WT bacteria with a genetically diverse or a derived clonal phage population. Each line represents a single replicate. **b** The average proportion of cells at 3 dpi that evolved CRISPR-based immunity (purple), surface modification (sm)-based resistance (orange), or that remained sensitive (green) and **c** the average number of spacers acquired by each CRISPR clone in response to infection with clonal (blue) or diverse (purple) phage. All graphs show averages and error bars represent 95% confidence intervals. Individual data points are indicated with dots, and numbers next to the dots indicate the number of replicates in which the same value was observed.

In order to study if and how phage genetic diversity impacts the frequencies of CRISPR and sm-based resistance that evolve in the bacterial population following infection, we performed an evolution experiment and monitored the phage population dynamics as well as the levels of CRISPR-based immunity observed following infection of WT PA14 with the clonal or more genetically diverse phage DMS3vir populations. Consistent with previous studies [16, 20], we found that phage titres increased in all replicates following infection of the WT strain, which is expected given that bacteria are initially sensitive to the phage. However, from 1 dpi onwards, phage titres started to decline until complete extinction at 6 dpi, with no clear difference between the diversified and paired clonal phage populations (Fig. 2a, ANOVA, F(1,22) = 0.02, *p* > 0.8). However, analysis of individual bacterial clones that were isolated at 3 dpi revealed clear differences in the frequencies of CRISPR-based immunity and sm-based resistance that had evolved following infection with genetically diverse or clonal phage populations (Fig. 2b, ANOVA, F(2,1) = 54.72, *p* < 0.0001). Specifically, we found that the frequency of sm-based resistance was higher in populations challenged with a genetically more diverse phage population than in populations challenged with the paired clonal population (Post hoc analysis with Tukey HSD, *p* < 0.0001), and this was associated with a corresponding decrease in the frequency of CRISPR-based immunity (*p* < 0.0001) (Fig. 2b). These data therefore show that increasing phage genetic diversity can cause a decrease in the frequency at which CRISPR-based immunity evolves and a corresponding increase in the frequency at which sm-based resistance evolves.

### Bacteria evolving CRISPR-based immunity against genetically diverse phage carry more spacers

**Fig. 3:**
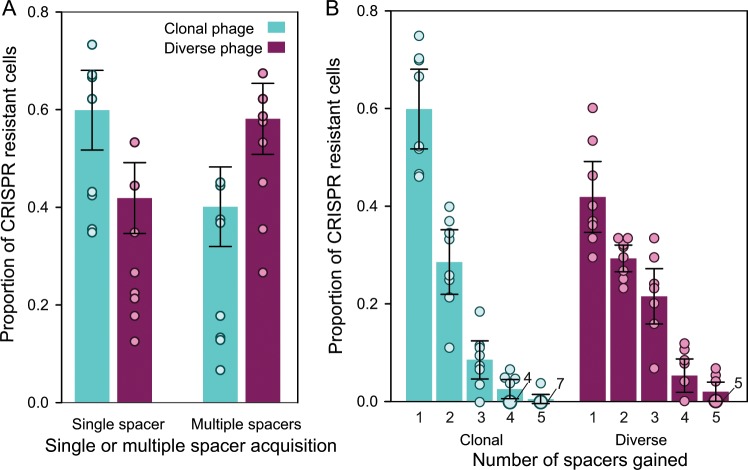
Patterns of spacer acquisition differ in response to infection with clonal or diverse phage. **a** Average proportion of CRISPR clones in a population which acquired single or multiple spacers when infected with clonal (blue) or diverse (purple) phage. **b** Average proportion of CRISPR clones in a population which acquired 1, 2, 3, 4 or 5 spacers when infected with clonal or diverse phage. All graphs show averages and error bars represent 95% confidence intervals. Individual data points are indicated with dots, and numbers next to the dots indicate the number of replicates in which the same value was observed.

Given that the number of spacers targeting a phage within a single host influences the propensity of phage to overcome CRISPR-based immunity (Fig. 1b and [16, 26]), we also examined how genetic diversity within infecting phage populations affects the number of spacers that individual bacterial clones acquire. PCR analysis of the CRISPR loci of bacterial clones following exposure to either the genetically more diverse phage DMS3vir populations or the paired clonal controls revealed that the patterns of spacer acquisition in cells using CRISPR-based immunity differed depending on the genetic diversity of the phage they were infected with (Fig. 3b). Specifically, upon infection with clonal phage the majority of cells which had acquired CRISPR-based immunity had acquired only one spacer (Fig. 3a, Tukey HSD, *p* < 0.005), whereas upon infection with genetically more diverse phage the majority of cells which had acquired CRISPR-based immunity had acquired multiple spacers (Tukey HSD, *p* < 0.05). Despite differences in the patterns of spacer acquisition, analysis with ANOVA revealed the average total number of spacers per clone gained was not affected (*F*(1,14) = 7.42, *p* > 0.1) (Fig. 2c). Collectively, the higher proportion of bacteria with sm-based resistance or CRISPR-based immunity with multiple new spacers in response to infection with genetically more diverse phage suggests selection for more generalist defence mechanisms under those conditions.

## Discussion

Here, we tested whether increasing genetic diversity within a parasite population will increase selection for generalist over specific immune defences. We find that infecting *P. aeruginosa* with experimentally evolved populations of phage DMS3vir that have elevated levels of standing genetic diversity results in, first, higher frequencies of sm-based resistance compared with CRISPR-based immunity and that, second, cells with CRISPR-based immunity are more likely to acquire multiple spacers. Interestingly no difference in phage persistence between populations that were infected with clonal or genetically more diverse phage populations were observed. These data show that genetic diversity has an important effect on the frequencies of CRISPR-based immunity (specialist defence) and sm-based resistance (generalist defence) in this system. While the elevated genetic variation of phage DMS3vir is presumably more reflective of natural environments compared with the clonal phage populations that are typically used in experimental evolution studies, it should be noted that our experiments do not capture the complexity of natural environments where different phages with overlapping host ranges tend to coexist, which can further impact the evolution of CRISPR-based immunity [31].

CRISPR-Cas is a specific and adaptive immune defence that is widespread, yet not equally abundant in different ecosystems. Although CRISPR-Cas systems are found in a diverse range of habitats, there is a highly uneven distribution across different environments. Metagenomic sequence analyses have shown that the frequency of the evolution and maintenance of CRISPR-Cas systems in bacterial and archaeal communities may be linked to environmental factors as higher frequencies of CRISPR-Cas are often found in extreme environments [18]. This suggests that there are environmental attributes that constrain the fitness advantages (or ecological success) of this form of phage defence. Some of the earliest studies that looked at the ecological distributions of CRISPR-Cas immune systems observed that these systems are overrepresented in high-temperature environments, with thermophiles typically possessing more and longer CRISPR arrays [32, 33]. Indeed, over 90% of hyperthermophilic archaea harbour CRISPR-Cas, and these environments are generally throught to be associated with moderate virus diversity [34]. The ancestral primitive CRISPR-Cas systems likely evolved in archaea and were subsequently acquired by bacteria through horizontal transfer [35]. It has been suggested that the prevalence of CRISPR-Cas in hyperthermophilic conditions may be due to the lower mutation rates in those environments, which is supported by correlational and theoretical studies [32, 36–39]. However, apart from accelerating the evolution of “escape” phage, high mutation rates will also increase the rate at which sm-based resistance evolves in the bacterial population, hence reducing the relative benefit of CRISPR-Cas immune systems [40]. Here we teased these two effects apart by exposing bacterial populations to experimentally diversified or clonal phage populations of the phage DMS3vir. Our experimental data support the idea that phage genetic diversity can tip the balance in the evolution of different defence strategies that bacteria employ to combat phage infections. Given that CRISPR-Cas immune systems are highly sensitive to the evolution of “escape” phage that overcome CRISPR immunity through mutation of their target sequences, greater levels of phage genetic diversity are likely to favour generalist defences that are more robust to phage evolution. Indeed, our experiments show that in this empirical system phage was unable to overcome sm-based resistance. Evolution of a novel receptor specificity would presumably require multiple adaptations in the phage tail fibres [24], and while this will occur in certain environments, the rate at which this happens will in most cases be low relative to the rate of CRISPR escape mutation.

The results of this study highlight how ecological variables can drive the evolution of bacterial defence strategies and it will likely be important to consider phage genetic diversity when studying natural systems. For example, we may expect to find higher levels of viral genetic diversity, and therefore lower levels of CRISPR-based immunity, in well-mixed environments such as in aquatic ecosystems, but the opposite may be true in systems with less migration, and therefore lower viral diversity, such as in soil. Environments associated with low viral mutation rates, as is often found in thermophilic microbial communities, will likely harbour higher levels of CRISPR-based immunity as generalist defences with a constitutive cost such as sm will have a selective disadvantage under these conditions. Biotic complexity is frequently ignored in laboratory evolution studies. It is also important to consider that in bacteria-phage interactions in natural ecosystems there will usually be a range of different phage types able to infect each host, and further work is needed to clarify how the presence of multiple phage would affect the evolution of bacterial resistance.

Here we have shown that diversity in an infecting parasite population can impact the outcome of host-parasite interactions. In the data presented here our experimentally evolved phages were passaged on a mutant genotype of the same host used in our experiments. Therefore, the mutations acquired may not be as varied as when phage evolves on a range of hosts as will typically be the case in nature. The observed effects of intraspecific diversity (i.e. genetic diversity within the same species of phage) have been previously predicted by studies that modelled CRISPR-phage interactions [36], but as far as we are aware this is the first work to demonstrate this experimentally. This work further contributes to our understanding of the role of the CRISPR-Cas adaptive immune system in a microbial community context, and complements previous work on the role of bacterial diversity [21]. Furthermore, this study suggests that genetic diversity is an important driver of when specific defences are favoured over generalist defences and should therefore be considered when investigating any host-parasite interaction where generalist or specific defences can evolve.

## Data Availability

Raw data files from the experiments have been uploaded to Dryad (10.5061/dryad.6djh9w0x7). Sequence data are available on the ENA PRJEB31472
